# TS-Resformer: a model based on multimodal fusion for the classification of music signals

**DOI:** 10.3389/fnbot.2025.1568811

**Published:** 2025-05-13

**Authors:** Yilin Zhang

**Affiliations:** Dalian University of Foreign Languages, International Art College, Dalian, China

**Keywords:** music genre classification, Fourier transform, residual network, transformer, attention mechanism

## Abstract

The number of music of different genres is increasing year by year, and manual classification is costly and requires professionals in the field of music to manually design features, some of which lack the generality of music genre classification. Deep learning has had a large number of scientific research results in the field of music classification, but the existing deep learning methods still have the problems of insufficient extraction of music feature information, low accuracy rate of music genres, loss of time series information, and slow training. To address the problem that different music durations affect the accuracy of music genre classification, we form a Log Mel spectrum with music audio data of different cut durations. After discarding incomplete audio, we design data enhancement with different slicing durations and verify its effect on accuracy and training time through comparison experiments. Based on this, the audio signal is divided into frames, windowed and short-time Fourier transformed, and then the Log Mel spectrum is obtained by using the Mel filter and logarithmic compression. Aiming at the problems of loss of time information, insufficient feature extraction, and low classification accuracy in music genre classification, firstly, we propose a Res-Transformer model that fuses the residual network with the Transformer coding layer. The model consists of two branches, the left branch is an improved residual network, which enhances the spectral feature extraction ability and network expression ability and realizes the dimensionality reduction; the right branch uses four Transformer coding layers to extract the time-series information of the Log Mel spectrum. The output vectors of the two branches are spliced and input into the classifier to realize music genre classification. Then, to further improve the classification accuracy of the model, we propose the TS-Resformer model based on the Res-Transformer model, combined with different attention mechanisms, and design the time-frequency attention mechanism, which employs different scales of filters to fully extract the low-level music features from the two dimensions of time and frequency as the input to the time-frequency attention mechanism, respectively. Finally, experiments show that the accuracy of this method is 90.23% on the FMA-small dataset, which is an improvement in classification accuracy compared with the classical model.

## 1 Introduction

With the popularization of the Internet and the development of artificial intelligence, the medium of music is no longer confined to records or tapes, but digital media such as cell phones, computers, mp3, and so on. Digital media and streaming platforms have provided strong support for the music industry, and a huge amount of music tracks have been made available to more and more people through diversified communication media, allowing them to access their favorite music anytime and anywhere. After 2015, the global music industry has entered the digital era. As of December 2023, China’s Internet penetration rate has reached 76.4% (Zhai and Luo, 2025). Thanks to the popularization of the Internet, the music industry has had better development. The global music streaming market size grew from about $15 billion in 2015 to about $43 billion in 2020, with a CAGR of about 23%. On this trend, the global music streaming market size will reach USD 82 billion by 2025. This growth trend is mainly driven by factors such as digitalized music consumption methods and the popularity of mobile internet. At the same time, competition among music streaming platforms is becoming more and more intense, with platforms constantly introducing new features and services to attract more users and increase user retention. In future development, music streaming media should not only provide high-quality audio services to attract more users but also apply artificial intelligence technology to introduce more intelligent recommendation systems and personalized services to improve user experience. There are various styles of music, and different genres have different audiences. Accurate identification of the genre for a new music track is a highly concerning issue, directly affecting the effectiveness of recommendation and user satisfaction. The traditional music genre classification method is mainly realized by manual classification, and the classifiers need to have high professional quality in music. Therefore, traditional music classification has the following drawbacks:

(1)   Not suitable for large-scale datasets.
(2)   The feature extraction method is complex and requires the designer to have a high level of expertise in music.
(3)   The feature extraction method lacks generalization, and different classification tasks need to compute music features separately and individually.

Therefore, there is a need for a music genre classification model with high accuracy, which utilizes the powerful arithmetic power of computers to achieve automated classification. In recent years, neural networks have made impressive achievements in image recognition, optimizing the tedious step of manually designing features in traditional machine learning and providing a new direction for music genre classification. Applying neural networks to the music genre classification task has much room for development, and deep learning technology provides theoretical support for it. The research on music genre classification methods based on deep learning mainly has the following problems:

(1)   The current research mainly analyzes the overall accuracy rate of all categories in the dataset, ignoring the lower accuracy rate of individual genre music classification.
(2)   Music has a strong time-series relationship, and the serial model architecture design has the problem of losing time-series information.

To address the above problems, we analyze and improve the model, and the main contribution consists of the following aspects:

(1)   For data processing, we design a cut-score method to cut music audio into different time durations. Experimentally, we analyze the effect of different time lengths on classification accuracy and training time, select the applicable cut time length for music, and realize data enhancement. The audio signal is converted to Log Mel spectrum by short-time Fourier change, Mel scale filter, and logarithmic compression. Compared with other features, the Log Mel spectrum fully preserves the characteristic information of the musical work by describing the energy intensity of the audio signal and the distribution of information in the time and frequency domains.
(2)   In terms of network model design, Res-Transformer, a parallel music genre classification model based on residual network and Transformer coding layer, is proposed, with the following design idea: unlike ordinary audio, music has a strong time-series relationship. Existing serial architectures use RNNs as temporal summarizers to extract time-series information, and their performance depends largely on the results of previous convolutional layers, and the time-series information of the original music is partially lost during the convolution process, resulting in unsatisfactory classification accuracy. To preserve the spatial characteristics and temporal order of the original music samples, the Transformer Encoder is used to extract the time series information of the music directly from the Log Mel spectrum. The RNN network predicts the frequency changes based on the neighboring time steps, while the Transformer’s multi-head self-attention layer enables the network to look at multiple previous time steps when predicting the next time step, which is better than the RNN. The effect is superior to RNN. Improve the residual network to enhance music genre feature extraction while avoiding gradient vanishing, add 1 × 1 convolution kernel in the jump connection to achieve dimensionality reduction, and introduce nonlinear transformations to increase the expressive power of the network.
(3)   In terms of model optimization, a parallel music classification model TS-Resformer is proposed based on the fusion of time-frequency and channel attention mechanisms in the residual network and Transformer coding layer. By adding the designed time-frequency attention mechanism in front of the residual module, the convolution adopts the different scales of filters to capture the features from the two dimensions of time and frequency, respectively. Experimenting with different scale convolution kernels for categories with low classification accuracy, the shapes of the two filters were analyzed and determined. After the time-frequency convolutional features are extracted and input into the time-frequency attention mechanism respectively, the feature map with time-frequency weights is formed by feature fusion.

## 2 Related work

Music is created by the inspiration of human beings, different combinations of instruments and different vocal choruses form a unique musical repertoire, and each song has a unique melody, rhythm, timbre, and other artistic elements, which are far different from ordinary audio. Music Genre Classification (MGC) is one of the branches of Music Information Retrieval (MIR), which is mainly used to classify different music genres.

As a classification task, Music Genre Classification mainly includes three steps:

(1)   Data preprocessing, which prepares for the next step of feature extraction by processing the original audio.
(2)   Feature extraction, which extracts the features that represent the information of music genres.
(3)   Classification, the extracted audio feature vectors are input into the classifier to realize music genre classification.

The results of traditional music classification scientific research are mainly reviewed from two perspectives: feature extraction and classification model.

In terms of feature extraction, traditional music classification extracts handmade features from the original audio signal. Arabi and Lu (2009) introduced chordal features in the process of feature extraction to better represent the characteristics of the music signal and combined them with low-level music features, using a support vector machine as a classifier, and experiments proved that combining low-level music features with high-level music features can improve the classification accuracy rate The paper is a comprehensive study of the classification of chord features in songs. The paper extracts chord features by counting the root notes of chords in each song, which is computationally complex and cannot realize end-to-end music classification. Logan (2000) proposed the Mel-Frequency Cepstral Coefficient (MFCC) and experimentally demonstrated the importance of the MFCC features in the field of audio recognition and the importance of the MFCC features in the field of music retrieval. And experimentally demonstrates the importance of MFCC features in the field of audio identification and its advancement and applicability in the field of music retrieval. Based on MFCC features, Baniya et al. (2014) used wavelet decomposition-based timbre texture (MFCC and other spectral features) and rhythmic content features to improve the performance. The paper retains the features that are useful for classification and discards those that are not effective, but there is a possibility of misclassification in the manual selection of features. Sarkar and Saha (2015) use empirical pattern decomposition to capture local features of different genres, and then compute pitch-based features from the decomposed songs, but the pitch features alone do not adequately characterize the musical features, and the method is only suitable for simple classification of music genres.

In terms of music classification models, Tzanetakis and Cook (2002) realized music classification by several models such as the Gaussian classifier, Gaussian mixture model (Duda et al., 2012) and K-nearest neighbor and the input features are hand-designed rhythmic content, pitch, and timbre features. This paper achieved the automatic classification of music genres earlier but did not design classification models for music features. Xi et al. (2004) extracted inverted spectral domain features of music, performed feature engineering to extract discriminative music features and achieved classification through Hidden Markov Models, but such models need to appropriately adjust the complexity of the model to avoid overfitting. Ali and Siddiqui (2017) used Principal Component Analysis to compare the performance of KNN vs. Support Vector Machines (SVMs) using Principal Component Analysis (PCA), and without dimensionality reduction, KNN and SVMs perform well, and SVMs are more efficient.

In addition, Sturm (2012) analyzed the GTZAN dataset, which is widely used in the field of music genre classification, and experimentally proved that this dataset has the problem of missing labels and errors, and the experimental results obtained on this dataset in the past are not accurate.

Deep learning models can achieve end-to-end learning, i.e., the complete learning process from the original data to the final prediction result, simplifying the design and optimization process of the system and improving the overall efficiency of the model. In recent years neural networks have gained great success in the fields of computer vision as well as speech recognition, and the field of music information retrieval has also begun to widely use deep learning neural networks (Orjesek et al., 2022; Ostermann et al., 2023) to realize end-to-end music genre classification (Solanki and Pandey, 2022; Lin, 2022). For deep learning music classification methods are mainly introduced in terms of feature extraction, network model, and attention mechanism.

In terms of feature extraction, researchers have investigated a variety of audio feature extraction methods. Pelchat and Gelowitz (2020) reviewed some of the machine learning techniques used in the field, utilizing spectrograms generated from song time slices as inputs to a neural network. Spectrograms contain information about multiple musical features, which facilitates model training. Chen et al. (2024) achieved 68.78% accuracy on the FMA-small dataset by using a CNN with residuals in series with bi-GRU, and Mel spectrograms as inputs were better compared to acoustic spectrograms obtained from short-time Fourier transform. Traditional MGC methods only consider audio information or lyrics information, which leads to unsatisfactory recognition results. Li et al. (2023) proposed a multi-modal music genre classification framework that integrates audio information and lyrics information. The framework uses a convolutional neural network to extract audio features from the Mel spectrogram while obtaining a distributed representation of the lyrics. The two modal information is fused through two different strategies, feature level, and decision level, but this approach increases the computation time.

Deep learning network models mainly utilize the strong recognition ability of convolutional networks on image information to achieve music genre classification. Zhang et al. (2016) proposed a CNN model that combines residuals with maximum-minimum pooling to provide higher statistical information for neural networks, but this model does not take into account the time-series information of the music. Choi et al. (2017) proposed a two-dimensional convolutional recurrent CRNN model the convolutional layer extracts features followed by a recurrent and fully connected layer to perform the classification task. This model is popular and many variants have evolved from it. Yang et al. (2020) parallelized CNN and RNN networks to process the inputs, allowing the RNN network to process the original spectrogram instead of processing the output of the CNN but suffered from insufficient feature information extraction. Vaibhavi and Krishna (2021) demonstrated the effectiveness of the CRNN in exploiting the spatial feature extraction capability of CNN and the RNN summarization time of the RNN in the GTZAN dataset. The capability of CNNs and the ability of RNNs to summarize temporal patterns (Verma et al., 2023). Kostrzewa et al. (2021) proposed multiple integrations of CNNs, RNNs, and CRNNs to combine the advantages of different deep neural network architectures, and evaluated this approach on the FMA-small dataset, obtaining an F1 score of 54.9%.

Incorporating the attention mechanism in the model and utilizing the attention mechanism to further improve the accuracy of music classification is one of the more popular research directions in recent years. Zhuang et al. (2020) proposed a method to achieve music classification using a Transformer classifier without using loop and convolutional structure and achieved 76% accuracy on the GTZAN dataset, which is not ideal. Prabhakar and Lee (2023) utilized a classifier with a graphical attention model to improve image processing, image attention network can automatically learn important regions and features in an image to improve the effectiveness of an image processing task. By weighting different regions in the image, the network can pay more attention to the important information. Khasgiwala and Tailor (2021) learned different levels of self-attention, i.e., learning to find relationships between different parts of the image by Vision Transformer X by retaining the positional information of the features in the image, comparing CNN, RNN-LSTM, Vision Transformer X on FMA. Wen et al. (2024) to solve the problem that the limited sensory field of a convolutional neural network cannot capture the correlation between the time frames of vocalization at any moment and the sound frequencies of all vibrations in the song, applied dual parallel attention to focus on global dependencies in CNN-5, proposed parallel channel attention to constructing the global time-frequency dependencies in the song, and designed the double parallel attention to focus on the global time-frequency dependence in songs. Many models do not effectively design the feature extraction layer of the convolutional structure for the music signal features, and the feature extraction part is relatively simple, which causes the models to neglect the extraction of local features. To solve the above problems, Xie et al. (2024) proposed a model using a one-dimensional res-gate CNN to extract local information of audio sequences. To aggregate the global information of audio feature sequences, the Transformer is applied to the music genre classification model, and the decoder structure of the Transformer is modified according to the task. Zhang et al. (2025) proposed and applied a novel talking face generation framework, termed video portraits transformer (VPT) with controllable blink movements. In the audio-to-landmark stage, the transformer encoder serves as the generator used for predicting whole facial landmarks from given audio and continuous eye aspect ratio (EAR). Zhang et al. (2024) proposed a convolutional dynamically convergent differential neural network (ConvDCDNN) to solve the classification problem of the electroencephalography (EEG) signals. First, a single-layer convolutional neural network is used to replace the preprocessing steps in previous work. Then, focal loss is used to overcome the imbalance in the dataset. After that, a novel automatic dynamic convergence learning (ADCL) algorithm is proposed and proved for training neural networks.

## 3 Methods

### 3.1 Music data preprocessing

In Section 3.1, first of all, we introduced and presented the dataset we selected. Then, we perform file integrity checking, data normalization, data augmentation and dataset splitting on the original audio, and then transform the audio file to generate the Log Mel spectrum as the input for the subsequent TS-Resformer model we proposed.

The most commonly used datasets for music genre classification tasks are GTZAN and the Free Music Archive (FMA) dataset. The dataset we use is FMA, a large music audio dataset designed specifically for MIR studies, which is larger and more specialized than the other datasets, providing 106,574 songs from 16,341 artists arranged in a 161-genre hierarchical structure and associated with dedicated metadata. It is categorized into three versions: large, medium, and small. Considering the computational resources, we adopt the small version, which has far more songs than the commonly used GTZAN dataset.

**Figure 1:**
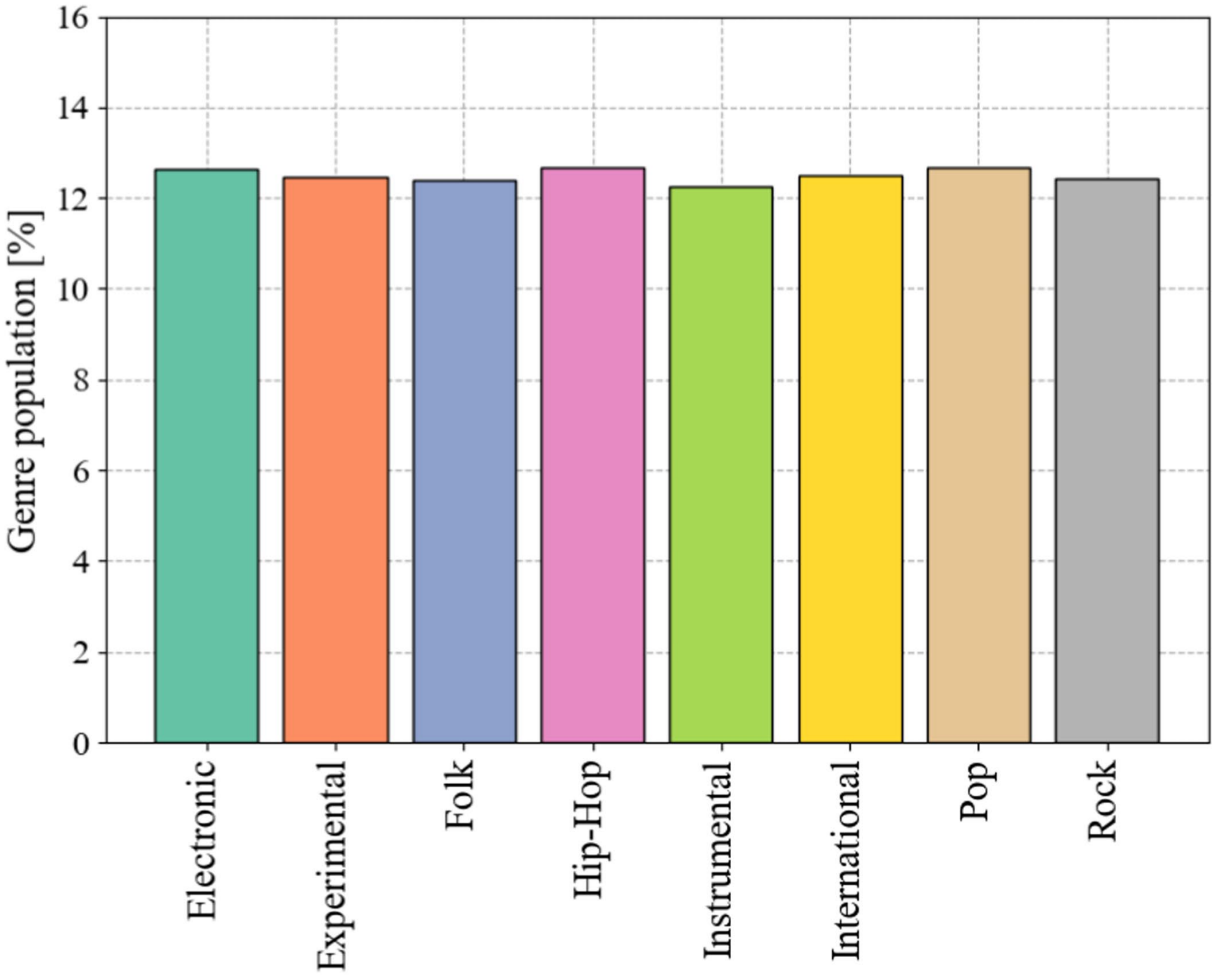
The proportion of eight musical analogies in FMA-small data set.

The FMA-small dataset contains a balanced subset of 8,000 songs distributed in 8 genres: Electronic, Experimental, Folk, Hip-Hop, Pop, Rock, Instrumental, and International. Each music clip is saved in map3 format, with a time length of about 30s and a sampling rate of 44,100 Hz. The number of music in each genre in the FMA-small dataset is shown in Figure 1.

To exclude interference from corrupted files as well as to improve classification accuracy, the following data processing steps are performed on the raw audio:

(1)   Verify track integrity

Corrupted audio tracks with unsatisfactory sampling rates and unsatisfactory time lengths are discarded to ensure that the audio files left behind can be recognized properly. The processed dataset has 7,992 music tracks.

(2)   Data augmentation

Addressing the issues of how varying audio durations affect the accuracy of music genre classification and training time, an approach of segmenting the original audio is adopted. Separate experiments are conducted to select an optimal audio duration suitable for the task of music genre classification. On the other hand, segmenting the original audio can achieve data augmentation, where more training data enhances the model’s generalization performance. The model designed for the music genre classification task should be suitable for large-scale datasets. In the FMA-small dataset, each excerpt *C* of a song’s genre has a duration of 30 s. Data augmentation is achieved by segmenting these music excerpts, with each resulting sub-segment *c_i_* having a duration of l seconds and a 50% overlap between two adjacent sub-segments. After segmentation, *N* sub-segments of l seconds each can be obtained, and each segmented sub-segment carries the same genre information as the original excerpt, defined as per the Equation 1:


(1) $$C=\{c_{1},c_{2},\cdots ,c_{n}\}$$

For example, slicing a Rock genre music clip in the dataset will result in 4 sub-clips of 12 s duration, each of which is labeled with the Rock genre.

(3)   Z-score normalization of raw audio

The Z-Score method calculates the amplitude mean of the audio by taking the absolute values of all the music amplitudes and then summing and dividing them by the number of samples. The Z-Score method normalizes the audio based on the mean and standard deviation of the original audio. Normalization is applied to the original audio to prevent numerical overflow, improve the stability of model training and convergence speed, and better adapt to the input requirements of the model, Z-Score the Equation 2.


(2) $$x\prime =\frac{x-\mu }{\sigma }$$

where *x* denotes the original data, *μ* denotes the mean of the original data, *σ* denotes the standard deviation of the original data, and *x*’ denotes the normalized data.

(4)   Slicing the data set

The dataset is sliced into training set, validation set, and test set according to 8:1:1, and the number of songs of different genres in the three sets is guaranteed to be balanced. The librosa library is utilized to load audio clips in each training selection generation.

**Figure 2:**
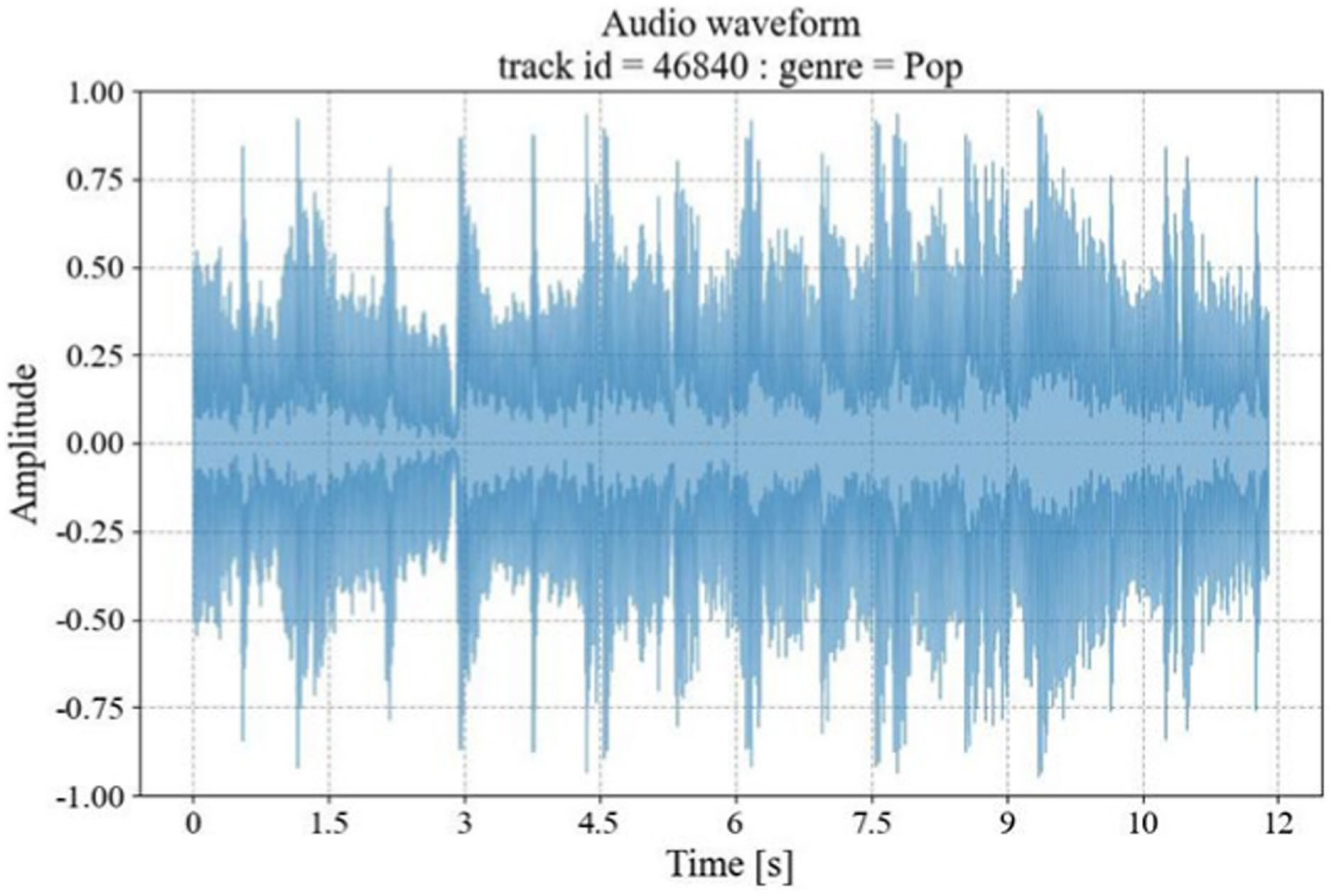
Original audio waveform diagram.

The audio waveform graphs show the amplitude changes of the audio signal at different time points, and the original audio waveforms are shown in Figure 2.

The horizontal axis of the graph is time, and the vertical axis is the sound amplitude; when the amplitude is larger, the greater the vibration amplitude of the waveform; a smaller amplitude means that the vibration amplitude of the waveform is relatively small.

Although the time domain can be converted to the frequency domain by Fourier transform, so that the frequency distribution expression is intuitive, the time domain information is lost. Therefore, the use of a short-time Fourier, wavelet time-frequency domain analysis method is more effective in avoiding such problems. We mainly use short-time Fourier transform to process the music audio. Through a series of processing methods, the audio is transformed into a Log Mel spectrum, which describes the energy distribution of the music at different times and corresponding frequency ranges, provides the conditions for the model to fully extract the audio features, and has the following advantages:

(1)   Distribution of time-frequency information

The Log Mel spectrum contains more information about music features, and the time-frequency distribution is clearer, which describes the change of the music signal frequency over time respectively, and uses the depth of the color in the third dimension to indicate the magnitude of the sound intensity in decibels (dB). The change of frequency peaks can be observed and contains more information about the music features compared to the traditional spectrogram.

(2)   Robustness

The Mel filter is used in the transformation process of the Log Mel spectrum, which filters the interference noise in the music audio to a certain extent and is less affected by the noise and environmental changes, which makes the accuracy of the experimental results increase.

(3)   Strong modeling capability

As the Log Mel spectrum contains several features such as Mel inverse spectral coefficient, logarithmic amplitude spectrum, etc., it can better characterize the spectral characteristics of the audio signal, and the calculation of the Log Mel spectrum is relatively simple, which only involves the calculation of the Mel filter bank and the operation of taking logarithmic numbers, whereas the MFCC needs to carry out the operation of the inverse spectral transform and the discrete cosine transform, etc. As the representation of the linear features, it is difficult to capture the complex structure and nonlinear features of the audio signal, and it is not easy to capture the complex structure and nonlinear features of the audio signal. As a linear feature representation, MFCC makes it difficult to capture the complex structure and nonlinear features of audio signals, while the Log Mel spectrum is closer to human auditory perception.

(4)   Easy to train the model

**Figure 3:**
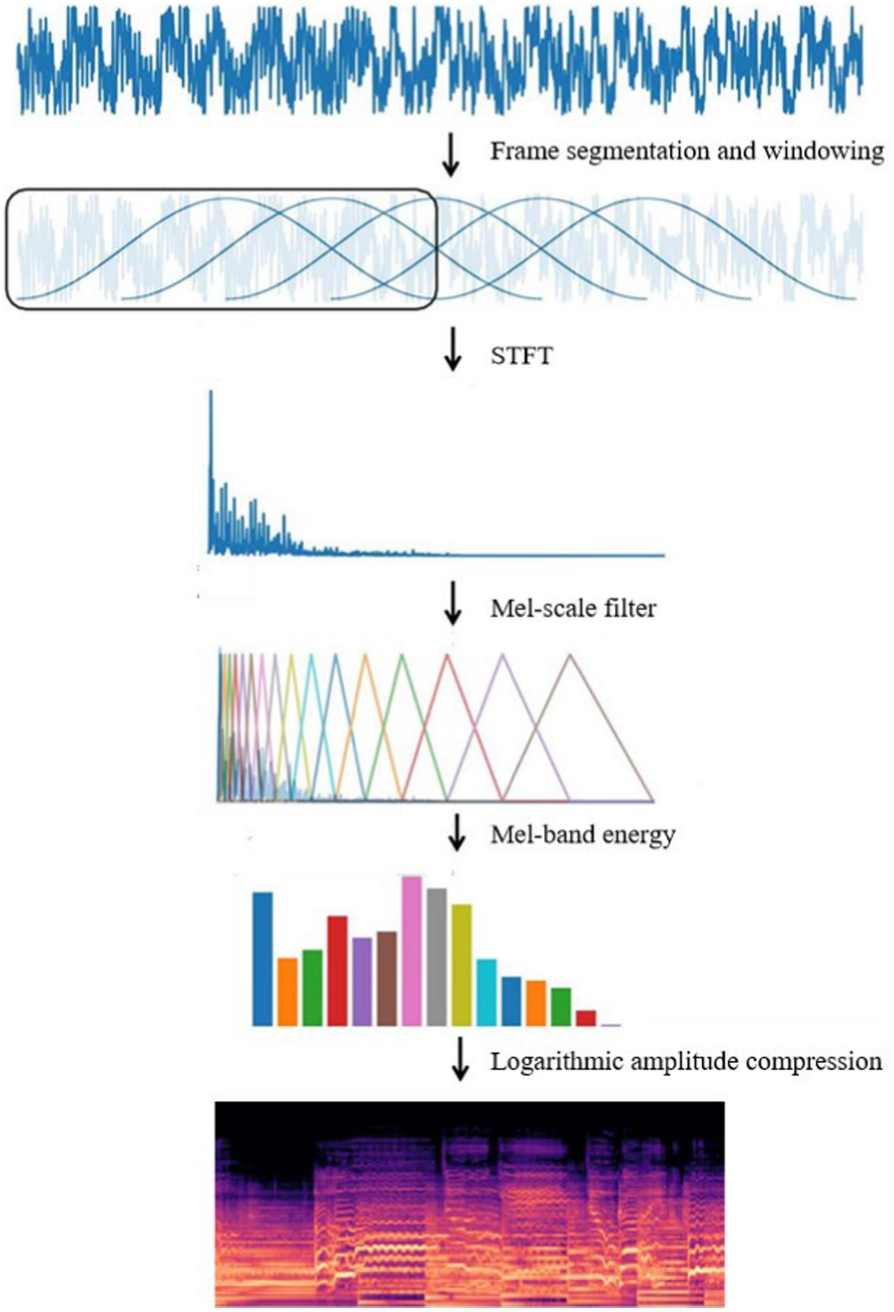
Log Mel spectrum conversion process.

Log Mel spectrum converts the audio signal into a two-dimensional image matrix, which reduces the input data of the model compared to the original audio form and makes the training efficient. Its form is similar to an image, and the neural network model is suitable for processing image data, and it is easy to be integrated with deep learning frameworks for end-to-end training. The Log Mel spectrum shows the frequency of the audio signal over time, and the flow of the conversion of the original audio to the Log Mel spectrum is shown in Figure 3.

In the music genre classification task firstly the pre-processed music audio is sub-framed and windowed. Music audio is a continuous signal that changes over time, and frame splitting divides the continuous audio signal into short time segments, each of which can be considered as a steady state. Split framing helps to capture the notes and pitch changes of music without affecting the temporal structure of the whole music. At the same time, the music can be considered frequency stable in short time segments, which helps to deal with large frequency variations in music audio, such as pitch changes of musical instruments. Windowing the audio after frame splitting reduces the risk of spectral leakage, and the windowing operation makes the music segments transition smoothly in time and reduces noise due to signal discontinuities.

The short-time Fourier transform is applied to each frame of the audio signal, based on which the energy of each Mel band is obtained by applying the Mel scale filter, and finally, the Log Mel spectrum is obtained after logarithmic compression, which expresses the music information in terms of time, frequency and energy intensity. The short-time Fourier transform the Equation 3:


(3) $$X(t,f)=\int_{-\infty }^{\infty }x(t)w(t-k)e^{-j2\mathrm{\pi fk}}dk$$

where *w*(*t*-*k*) is the time window function, multiplied by the signal *x*(0) for Fourier transform, and the spectral operation the Equation 4.


(4) $${\mathrm{SP}}_{x}(t,f)=|X(t,f)|2=|\int_{-\infty }^{\infty }x(t)w(t-k)e^{-j2\mathrm{\pi fk}}dk|2$$

The short-time Fourier transform is a key step in the process of transforming the Log Mel spectrum from audio, and the choice of window length in the short-time Fourier transform directly affects the experimental results. We experimentally created these audio clips using a window containing 4,096 samples, with a jump window size of 1,024, and used the Hanning window function to reduce the amplitude of the discontinuities at the boundaries, which is close to 1 s for 4,096 samples in terms of duration. The window size is chosen to be a power of 2 to ensure the high efficiency of the FFT algorithm, and if this is not the case, a zero-completion operation can be used. In addition, using 4,096 samples as the window size ensures that the FFT is performed with uniform frequency resolution and reduces the occurrence of spectral leakage. Spectral leakage refers to the deviation of the signal frequency waveform due to the truncation of the window function, which leads to a reduction in the accuracy of spectral estimation.

The human ear perceives low-frequency tones and high-frequency tones nonlinearly, with low-frequency tones being low and thick and high-frequency tones being sharp. The human ear perceives mid-frequency tones, which are the dominant frequency range of most human voices and musical instruments, more strongly. The Mel filter bank simulates the nonlinear perception of sound frequencies by the human ear through the analog Mel scale. The Mel filter bank divides the frequency spectrum into frequency bands by the auditory characteristics of the human ear, and each band corresponds to the energy response of a Mel filter. Therefore, converting the audio signal from a linear frequency to the Mel frequency scale better reflects the way the human ear perceives sound. Mel spectrograms are created by applying a set of 128 overlapping Mel scale filters to calculate the spectral energy of each frequency band, and each Mel spectrogram has the shape of [128, 512], which fully preserves the characteristic information of the music and reduces the dimensionality of the model input data.

The Mel scale conversion the Equation 5:


(5) $$\mathrm{Mel}(f)=2595\cdot \log_{10}(1+\frac{f}{700})$$

**Figure 4:**
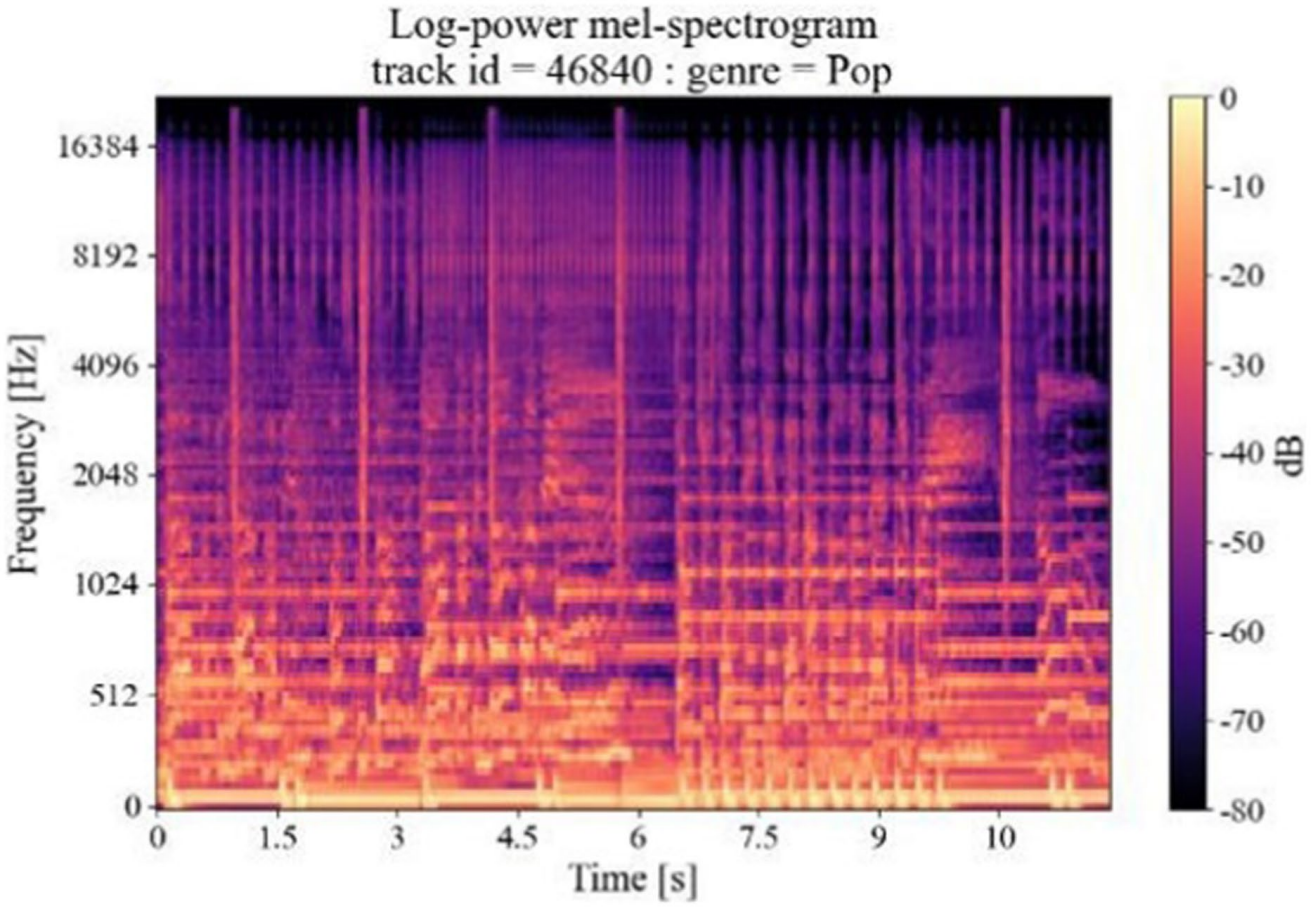
Log Mel-spectrogram.

The amplitude of the Meier spectrogram is converted to a decibel (dB) scale, which compresses the dynamic range of the signal and makes it easier to identify the relative strengths of the different frequency components of the spectrogram. The logarithmic Mel Spectrogram obtained from the conversion of the audio clip waveform graph shown in Figure 2 is shown in Figure 4.

### 3.2 TS-Resformer model

#### 3.2.1 General structure

**Figure 5:**
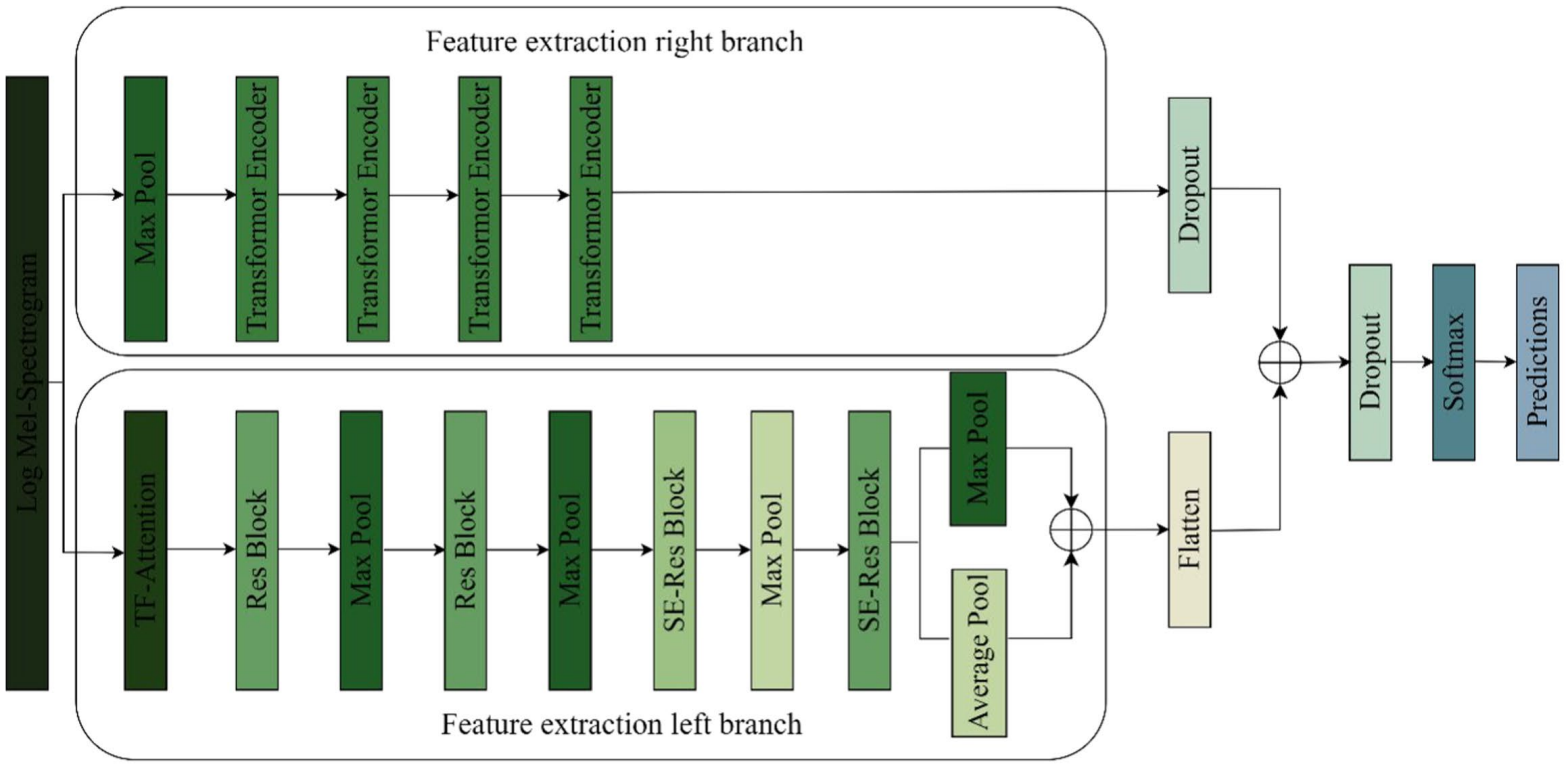
TS-Resformer model network architecture.

We propose an improved residual network and transformer encoder parallel music type classification model-TS-Resformer. The model structure is shown in Figure 5. First, we parallelize the residual network with the Transformer Encoder module, the left branch of the feature extraction is the residual network to extract the spectral feature information, and the right branch of the feature extraction is the four-layer Transformer Encoder to extract the time series information. Then, different scales of filters are designed for the four types of music genres with poor left classification effect, focusing on extracting time domain and frequency domain features respectively, fully extracting the time-frequency features and designing a new time-frequency attention mechanism to be applied on the branch. Finally, to enhance the model’s attention to important features between channels and capture the spatial relationship between features, the residuals are combined with the SE Block to form a new SE-Res Block.

#### 3.2.2 TF-attention module

**Figure 6:**
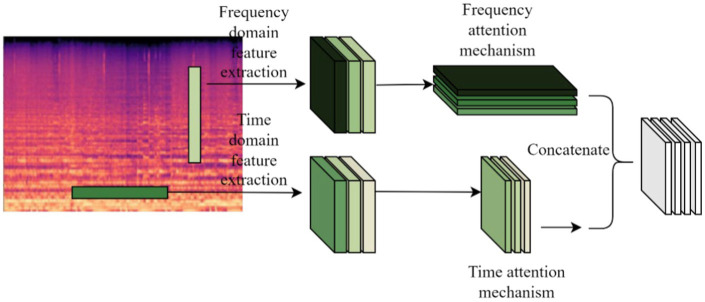
TF-attention module.

Most attention-based MGC systems add an attention mechanism at the end of deep learning network architecture feature extraction to learn high-level musical representations (Zhang et al., 2025; Zhang et al., 2024). Existing time-frequency attention mechanisms mainly extract high-level features at the bottom of the model, but low-level musical features such as energy, pitch, and tone are not sufficiently extracted, limiting the performance of the MGC task. Therefore, the time-frequency attention is placed after the multi-scale time-frequency feature extraction layer Conv1, and the extracted time-frequency features are learned, and the TF-Attention module, which integrates the time-frequency attention mechanism with time-frequency feature extraction, is shown in Figure 6.

**Figure 7:**
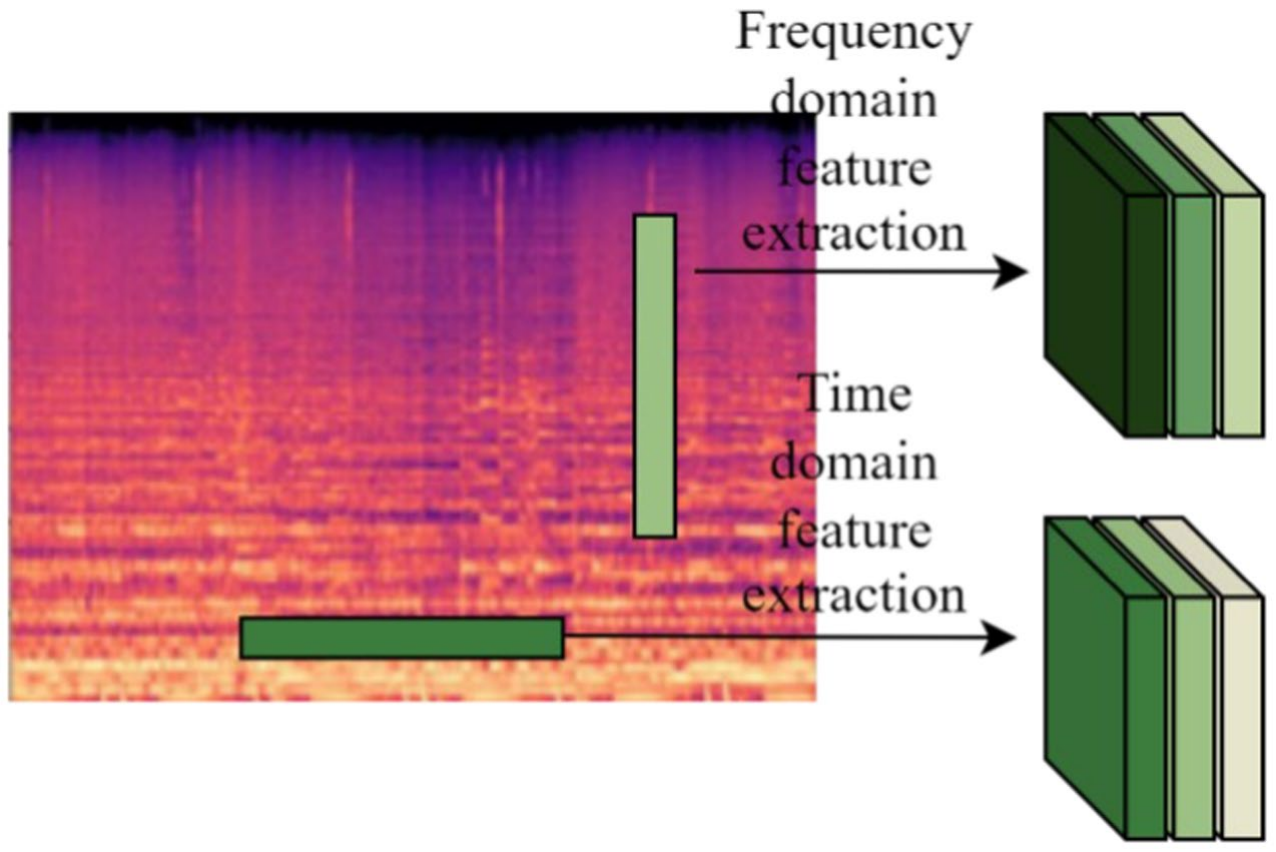
Time-frequency feature extraction.

We designed the time-frequency feature extraction layer Conv1 applicable to Log Mel spectrums to extract more features from Log Mel spectrums. Two parallel convolutional filters with different shapes are designed to learn the feature representation across time and frequency as shown in Figure 7.

The data in the Conv1 layer represents input channel 1 and output channel 32 respectively, the filter shape in the frequency domain feature extraction part is *M* × 2, and the filter shape in the time domain feature extraction part is 2 × *N*. Music is quite different from normal audio, so the shape of the two parallel convolutional filters needs to be re-determined, i.e., the values of *M* and *N* need to be determined experimentally. Finally the extracted features from the two branches are fed into the lower network.

**Figure 8:**
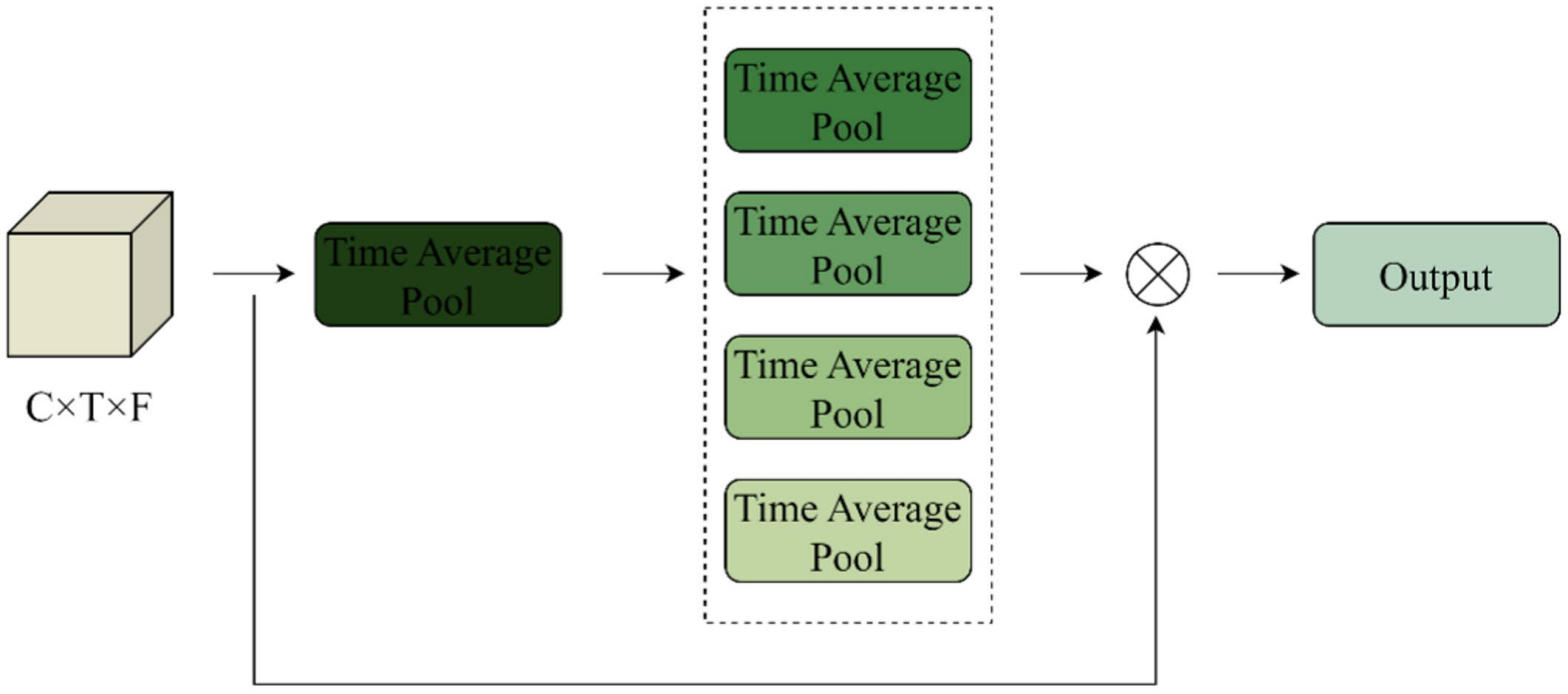
Time attention mechanism structure diagram.

Conv1 inputs the extracted music time-frequency features into the Time-Frequency Attention Mechanism module to mine effective low-level music features and assigns corresponding weights to the input audio in the time and frequency domain directions to form a feature map with time-frequency weighting information, learns the important information, and discards the information that is irrelevant to music classification. The internal structure of the time domain attention mechanism is shown in Figure 8.

The frequency attention mechanism is similar. The input is 1x128x512, and the feature model is generated after average pooling of X along the time direction, *Y_t_* ∈ *R*^1*1*T^, operating as the Equation 6.


(6) $$Y_{t}=\frac{1}{T}\sum_{n=1}^{T}X(n)$$

The scaling of the first convolutional layer is set to 16, the ReLU activation function is used to process it, and then a layer of convolution is used to construct the inter-channel correlation, and finally the Sigmoid is used to fix the value between 0 and 1 to get the weight in the time direction, which is multiplied by the original input to get the new feature map with the time weights. Similarly, the feature map with frequency weights can be obtained, and the two new feature maps are spliced together and fed into the lower layer network for processing.

Using Sigmoid as a scaling function for audio signals avoids focusing attention on a few time frames. By selectively aggregating the front and back frames according to the time-frequency attention, similar musical features achieve a common facilitation, thus improving the compactness within the class. Channel concatenation has advantages: first, the lower layers are able to receive large regions in the time and frequency domains, providing more information for advanced representation learning.

#### 3.2.3 SE-res module

Log Mel spectrums generated after the feature extraction layer of the time-frequency attention mechanism have a better representation of the music, but also do not classify well if the model does not focus well on important feature information. The SE module weights each channel of the feature map and effectively ignores features that are not relevant to the music classification in order to selectively emphasize the features that are more relevant to the music classification. The feature maps are extracted from the Log Mel spectrums, and the weights of each of these channels are dynamically adjusted by the channel attention mechanism to make the network model more representational. The channel attention mechanism consists of two steps: compression and excitation.

**Figure 9:**
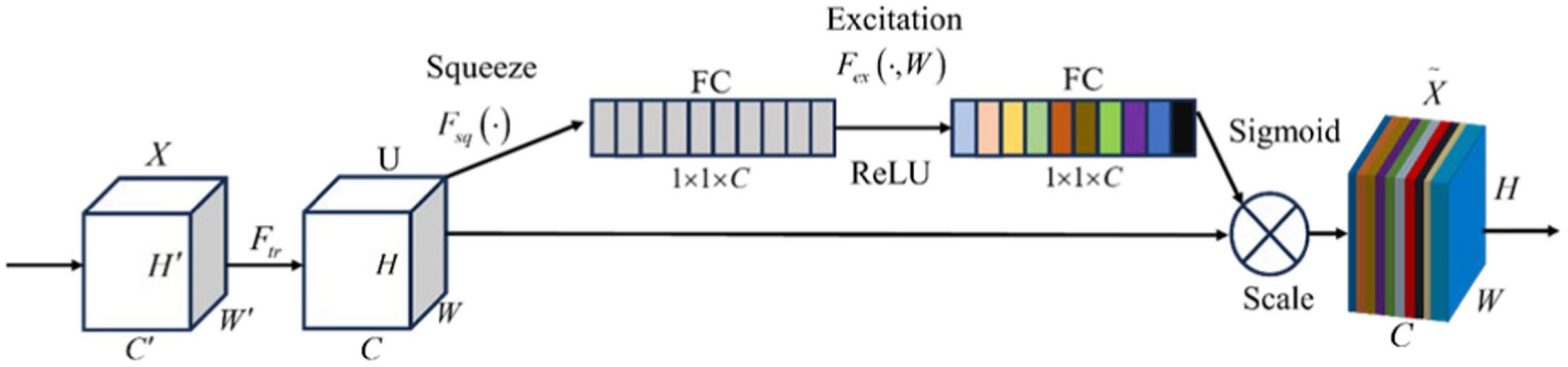
Squeeze-and-excitation module structure.

The structure of the SE module is shown in Figure 9.

The compression step compresses the feature map *U*∈*R*^*H* × *W* × *C*^ to 1x1x*C* by means of a global average pooling operation to achieve the conversion of spatial features to global features, and the resulting global information *z* is used as the feature weights reflecting the global importance of each channel, i.e., the degree of contribution of each channel to a particular task. The compression step is specified by the Equation 7.


(7) $$z_{c}=F_{\mathrm{sq}}(u_{c})=\frac{1}{H\times W}\sum_{l=1}^{H}\sum_{j=1}^{W}u_{c}(i,j)$$

where *u_c_* ∈ *R*^*H* × *W*^ represents the *c*th channel of *U*.

In the excitation step, the SE attention mechanism learns the weights of each channel through a fully connected layer. The network receives global features from the compression step as input and outputs a vector of channel attention weights. The specific the Equation 8 for the excitation operation:


(8) $$s=F_{\mathrm{ex}}(z,W)=\sigma (g(z,W))=\sigma (W_{2}\delta (W_{1}z))$$

where vector *s* represents the importance level of each feature map, *δ* represents the ReLU function, *σ* represents the Sigmoid activation function, $W_{1}\in R^{\frac{c}{r}\times c},\;\;W_{2}\in R^{c\times \frac{c}{r}}$ , and *r* is the scaling ratio, and the vector *s* is multiplied by the original feature maps to weight each channel, as specified in the Equation 9:


(9) $${\tilde{X}}_{c}=F_{\text{scale}}(u_{c},s_{c})=s_{c}u_{c}$$

He and Jiang (2021) built an SE-ResNet model for bone age assessment of hand X-ray imaging, so that the model would not lose important information in raw images. Kong et al. (2023) built the SE-ResNet model to form a complete glomerular classification framework to classify glomerular lesions. Liu et al. (2024) built an SE-ResNet model to classify heartbeat between patients, so that the model can effectively learn the long-term characteristics of heartbeat between patients. It can be seen from the above literature that in different fields, the authors combine SE module with ResNet model, so that the classification ability of the model gets a good effect. Therefore, we apply this combination to music signal classification, in order to make the model pay better attention to the important feature information in the Log Mel spectrum and pay better attention to the long distance feature information in the Log Mel spectrum.

**Figure 10:**
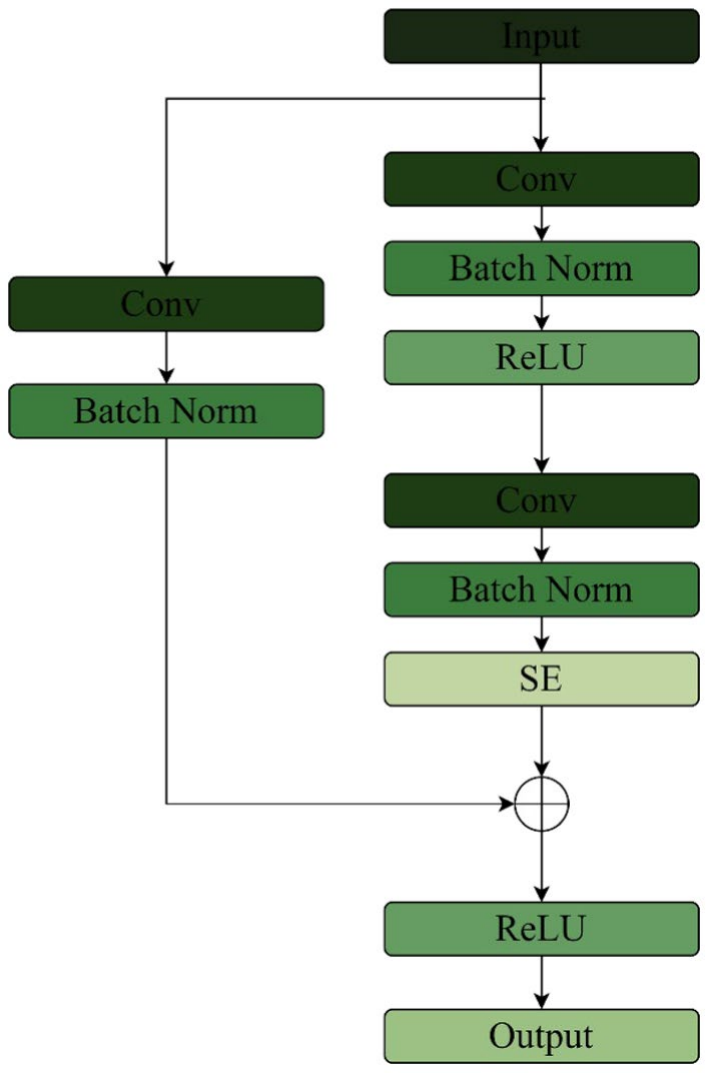
SE-res module structure.

The SE layer is placed after the two convolutional layers of the Res Net backbone network, and the jump connections are connected to the output of the SE. The improvement is to replace the fully connected layer in the SE with a 1 × 1 convolution. Firstly, the convolutional layer learns and adjusts the channel attention by sharing parameters. Secondly, the 1 × 1 convolutional layer retains the spatial information when feature extraction is performed on the feature map, and the attention weight of each channel can be adjusted with the spatial information, which is conducive to capturing the spatial relationship between features better. The fully connected layer, on the other hand, spreads the input into vectors without considering the spatial structure of the input Log Mel spectrum and loses the spatial information, so it is not suitable for the processing of the Log Mel spectrum. Finally the convolutional layer has the properties of local connectivity and parameter sharing, which can simultaneously process input data at multiple locations and improve the model generalization ability. The structure of SE-Res module is shown in Figure 10.

#### 3.2.4 Transformer encoder module

Transformer’s multi-headed self-attention layer is able to look at multiple previous time steps when predicting the next time step, and the model is able to capture dependencies over longer distances. The music genre classification task needs to take into account longer contextual information, such as the connection between the intro and chorus parts. Transformer obtains the positional information of the input music sequence through positional encoding, which allows the model to distinguish between different positional elements, with different timesteps representing different pieces of music. Compared to RNN networks, Transformer’s property of establishing connections over the entire range of sequences is more suitable for the music genre classification task.

**Figure 11:**
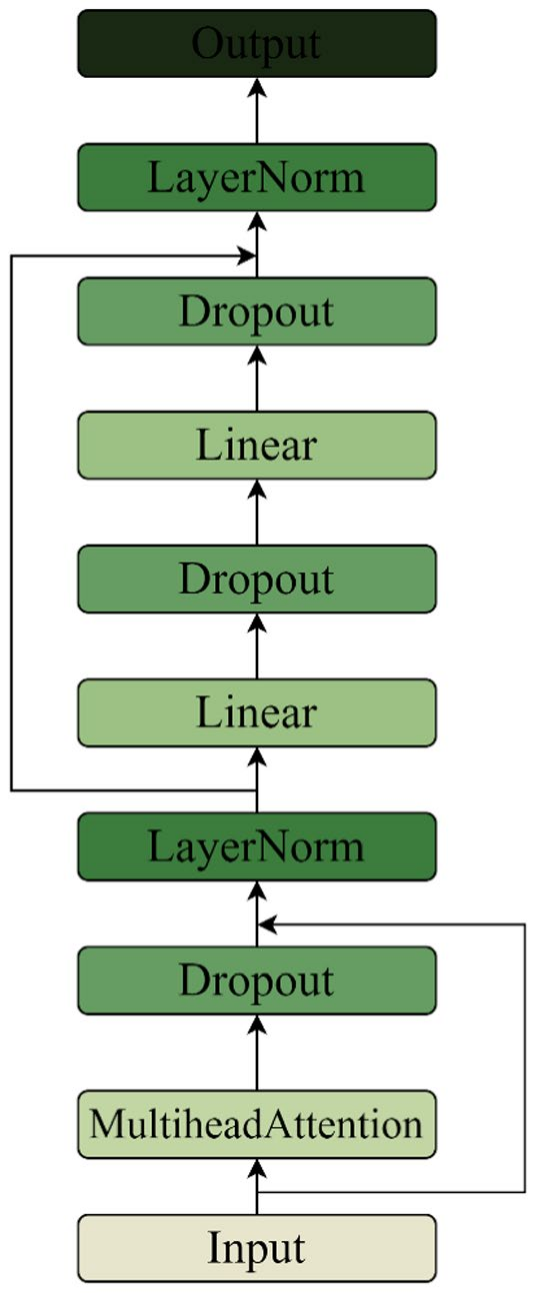
Transformer encoder module structure.

CNNs have excellent performance in extracting spectral features of music, but the network design of a CNN concatenated with an RNN loses time-series information during the extraction of Log Mel spectrum features. Therefore, a parallel Transformer module is used to complement the extraction of time-series information from Log Mel spectrums, while avoiding the problem of time-series information loss in CNN and RNN models. Each encoder block has 4 self-attention layers in each multi-head self-attention layer, and each encoder block has 2 linear layers in the feed-forward network. The Feed Forward is set to 512, and the Drouput is set to 0.4. The structure of the Transformer Encoder is shown in Figure 11.

## 4 Experiments

### 4.1 Experimental environment

The TS-Resformer model we designed is built on the PyTorch framework in Python3.8 environment. Comparison experiment and ablation experiment are also implemented in PyTorch framework based on Python3.8 environment. The detailed experimental environment is shown in Table 1.

**Table 1:** Experimental environment.

	Environment configuration
Operating system	Windows 10
CPU	AMD Ryzen 95900HX with Radeon Graphics 3.30 GHz
GPU	NVIDIA GeForce GTX 3080 CUDA 12.1

In the design process of TS-Resformer model, we selected the Adam optimizer, set the learning rate to 10^−3^, set the Batch size to 32, took the multi-class cross-entropy loss function as the loss function, and ReLU as the activation function. As for data preprocessing, we adopted the music data preprocessing method in Section 3.1, applied short-time Fourier transform to each processed music audio segment, the window function was Hanning window, the window size was 4,096, the jump length was set to 1,024, and the slice length was 12 s, with 50% repeat segments. The audio is processed according to the method described in Section 3.1, the Log Mel spectrum is generated, and the Log Mel spectrum is input into the TS-Resformer model for subsequent classification tasks.

### 4.2 Evaluation metrics

We choose the accuracy rate, confusion matrix and F1- Score as the evaluation indexes of the experiment, and use them as the basis for comparing the experimental effects.

Accuracy rate refers to the proportion of correctly predicted samples to the total number of samples by the classifier, and the formula for calculating the accuracy rate is shown below and the Equation 10:


(10) $$\text{Accuracy}=\frac{1}{k}\sum_{i=1}^{k}\frac{{\mathrm{TP}}_{i}+{\mathrm{TN}}_{i}}{{\mathrm{TP}}_{i}+{\mathrm{TN}}_{i}+{\mathrm{FP}}_{i}+{\mathrm{FN}}_{i}}$$

where *TP_i_*, *TN_i_*, *FP_i_*, *FN_i_*, are True Positive, False Positive, False Negative, False Negative, respectively, and the variable k denotes the number of classes. Since the dataset in this paper is balanced, macro-averaging is used, a method that calculates the metric for each class and then averages it, assigning the same weight to each class.

Precision is the ratio of the number of samples correctly predicted as positive classes to the number of all samples predicted as positive classes. It measures the proportion of samples that are truly positive categories out of all samples predicted as positive categories by the model, i.e., the accuracy of the model’s predictions. The formula for calculating the accuracy rate is shown below the Equation 11 for:


(11) $$\text{Precision}=\frac{1}{k}\sum_{i=1}^{k}\frac{{\mathrm{TP}}_{i}}{{\mathrm{TP}}_{i}+{\mathrm{FP}}_{i}}$$

Recall is the ratio of the number of samples correctly predicted to be in the positive category to the number of samples in all true positive categories, and measures the degree of coverage of the model for samples in the positive category, i.e., the model’s ability to recognize samples in the positive category. The Equation 12 for recall is shown below:


(12) $$\text{Recall}=\frac{1}{k}\sum_{i=1}^{k}\frac{{\mathrm{TP}}_{i}}{{\mathrm{TP}}_{i}+{\mathrm{FN}}_{i}}$$

F1-Score is the reconciled average of precision and recall, which is a comprehensive evaluation of the performance of the classification model, and is a weighted average of precision and recall, which can consider the accuracy and coverage of the model at the same time. The formula for calculating the F1-Score is shown below The Equation 13 for:


(13) $$F1-\text{Score}=2\frac{\;\text{precision}\times \text{recall}\;}{\;\text{precision}+\text{recall}\;}$$

The confusion matrix is shown in Table 2.

**Table 2:** Confusion matrix.

	Real positive results	Real negative results
Predicting positive outcomes	TP	FP
Predicting negative outcomes	FN	TN

### 4.3 Analysis of experimental results

#### 4.3.1 Training process

**Figure 12:**
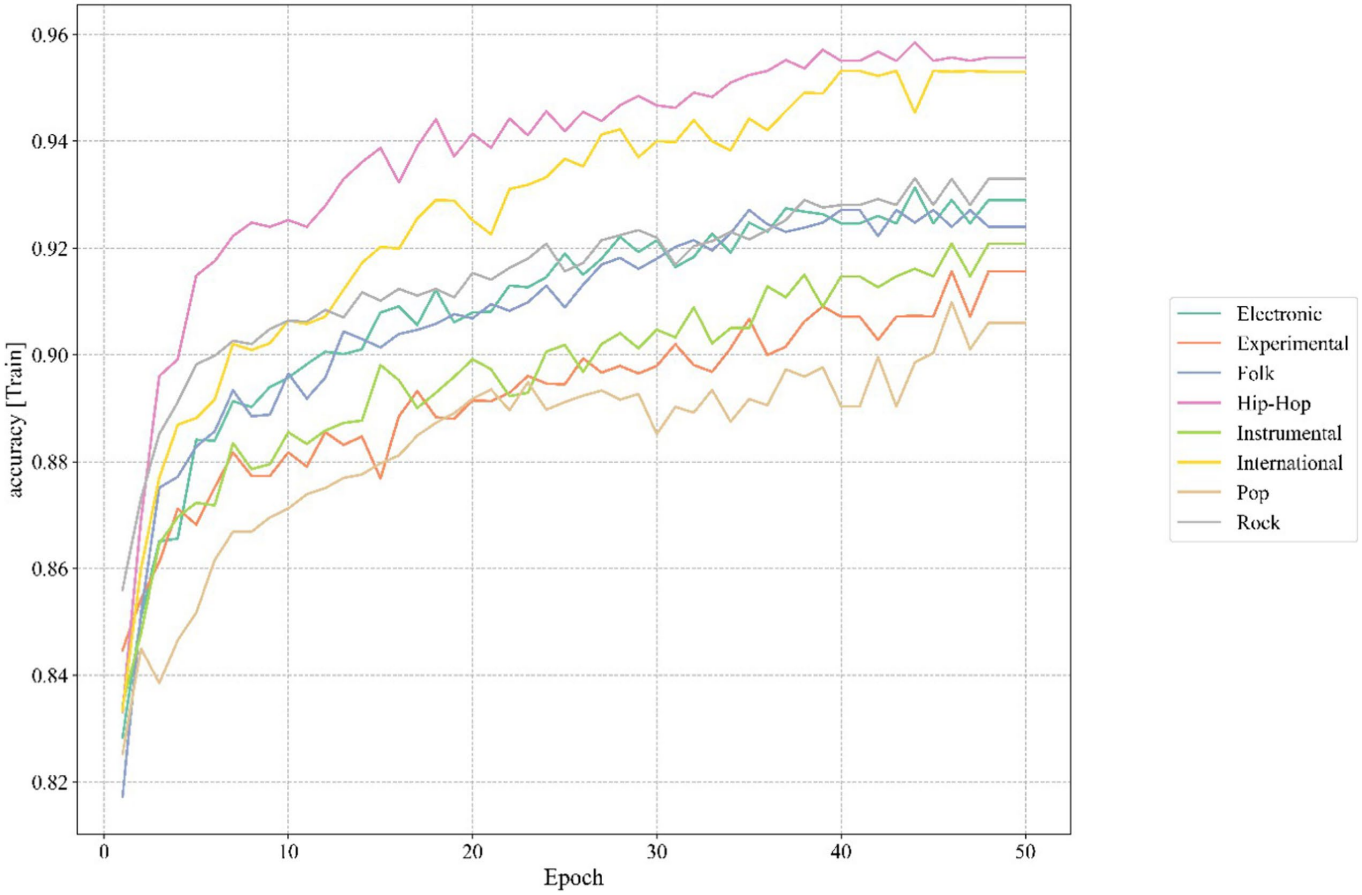
Training process.

The classification accuracy of the TS-Resformer model in each school of thought on the training set is shown in Figure 12.

It can be seen from Figure 12 that during the training process of our TS-Resformer model, around the 46th round, the accuracy rate of the model for various music categories has tended to be stable. This indicates that the model we proposed can converge at a relatively fast speed and achieve satisfactory results.

#### 4.3.2 The effect of different slicing lengths of music on model classification accuracy and training time

We design the audio duration slicing method with overlap rate, slicing the Equation 14.


(14) $$N=\frac{L}{\mathrm{\lambda I}}-1$$

Where *N* is the number of segments obtained by slicing an audio segment, *L* is the length of the original audio, the datasets used in this paper are all 30s segments, so *L* is 30s. *λ* is the overlap rate, which is set to 50% in this paper. *i* is the length of the segment obtained by slicing, and the complementary zero operation is performed for the case of insufficient sampling points. According to this formula, 30s audio can be sliced into [3 s,5 s,6 s,10s,12 s,15 s] according to the 50% overlap rate, and the corresponding number of segments is (Choi et al., 2017; Orjesek et al., 2022; Ali and Siddiqui, 2017; Sarkar and Saha, 2015; Baniya et al., 2014; Logan, 2000).

**Figure 13:**
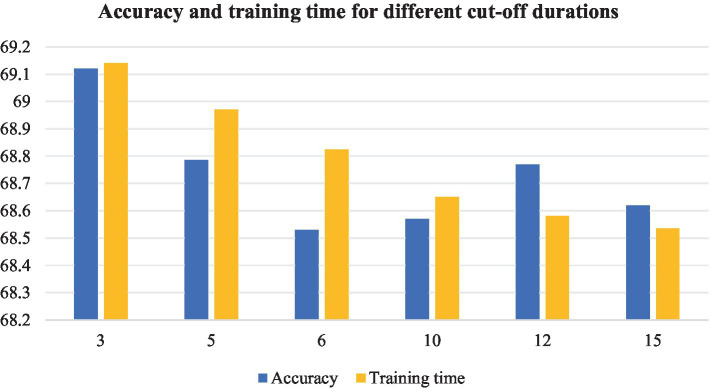
Accuracy and training time of different segmentation duration.

The short duration of the segment leads to insufficient feature extraction, and the whole audio input significantly increases the computational cost and affects the model design. In order to verify the effect of slicing audio segments of different durations on the experiments, experiments are conducted on the FMA-small dataset with the above audio slicing method. The experimental results are shown in Figure 13.

As the slice length increases, the total number of slices decreases and the training time decreases. a slice length of 3 s has higher accuracy, but makes the dataset extremely large, the training time the longest, and requires more equipment. As the slice length increases, the training time decreases. In the case of 12 s slice length, the accuracy is 0.36% different from the 3 s case.

Therefore, considering the above, we use 12 s to slice the 30s audio file into 4 segments with 50% overlap.

#### 4.3.3 Multi-scale convolutional filter shape determination

**Figure 14:**
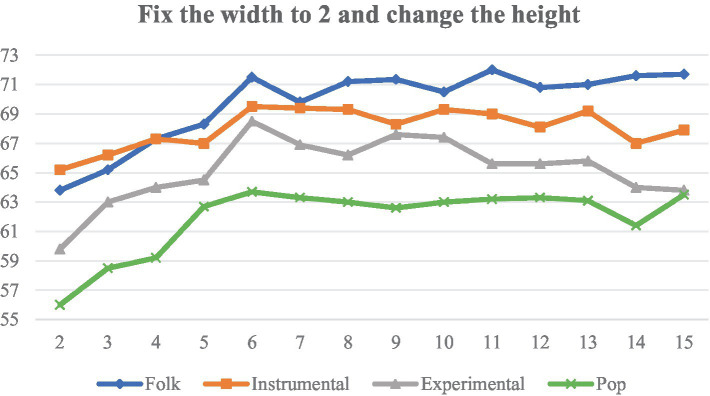
Classification accuracy of different filter heights.

On the FMA-small dataset, we selected four music genres with poor classification effects to carry out experiments to explore the effect of different shapes of filters on the classification effect under the same experimental environment, and the evaluation index of the experiments is the accuracy rate. Firstly, the filter width is fixed at 2, and the height of the filter is adjusted between 2 and 15, and the experimental results are shown in Figure 14.

**Figure 15:**
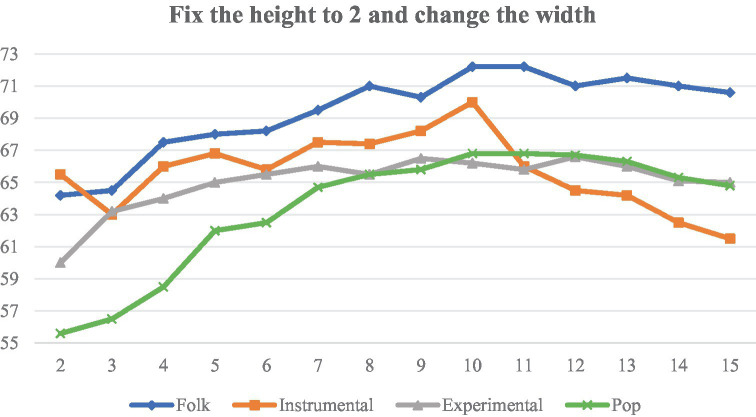
Classification accuracy of different filter widths.

Then the height of the filter is fixed to 2, and the width of the filter is adjusted between 2 and 15, and the experimental results are shown in Figure 15.

Figures 13, 15 are analyzed as follows:

(1)   As the height (frequency) increases, the accuracy of the four categories also increases. The overall trend flattens out at a height of about 6, beyond which further increases in frequency range do not result in significant improvements.
(2)   When the width (time) is increased, the sensory field time span becomes larger, and Instrumental first increases to a peak and then decreases rapidly, suggesting that Instrumental is expressed by short-term characterization, and that the overall accuracy is highest at a width of 10.

In summary, we chose 6 × 2 and 2 × 10 filters to apply to the Conv1 layer of the TF-Attention module.

#### 4.3.4 Ablation experiments

The classification accuracy of our proposed time-frequency attention mechanism, SE-Res module, and different network architectures on the FMA-small dataset is verified by ablation experiments, the results of which are shown in Table 3.

**Table 3:** Ablation experiments.

	Serial structure	Parallel structure	TFA	SE-res module	Accuracy	*F*1 *score*
1	√				87.64%	0.565
2		√			88.65%	0.593
3		√	√		90.16%	0.612
4		√		√	89.92%	0.603
5		√	√	√	90.23%	0.647

As can be seen from Table 3, by comparing 1 and 2, in terms of the two evaluation metrics of accuracy and F1-Score, the parallel structure we use is higher than the serial structure by 1.01% and 0.032, respectively, which indicates that the feature extraction of the parallel structure is more adequate than that of the serial structure, so that the model acquires sufficient features and improves the accuracy. By comparing 2 and 3, in terms of accuracy and F1-Score, the addition of our proposed TF-Attention module improves 1.51% and 0.019 respectively, indicating that the TF-Attention module further enables the model to focus on the main feature information in both the time and frequency domains. By comparing 2 and 4, the proposed SE-Res module improves the accuracy and F1-Score by 1.3% and 0.01 respectively, which indicates that the SE-Res module further focuses the model on the global feature information and improves the model’s expressive ability. In order to verify the rationality of selecting four Transformer encoder layers for our model, we conducted ablation experiments. We selected a different number of Transformer encoder layers for the model. A is a model containing one Transformer encoder layer, B is a model containing two Transformer encoder layers, C is a model containing three Transformer encoder layers, D is the model proposed in this paper, and E is a model containing five Transformer encoder layers. The experimental results are shown in Table 4. The experimental results show that the accuracy rate increases as the number of selected layers increases. When the number of layers increased to 4 layers, the accuracy reached the peak, and continued to increase the number of layers, the accuracy declined. According to the experiment, when the model chooses 4 Transformer encoder layers, the model has the best music signal classification ability.

**Table 4:** Ablation experiments.

	Number of layers	Accuracy
A	1	87.92%
B	2	88.25%
C	3	89.69%
D	4	90.23%
E	5	89.93%

#### 4.3.5 Comparison experiment

In this experiment, we evaluate the performance of several classical network structures and their combinations on the FMA-small dataset, including LSTM, GRU, Bi-GRU, ResNet, Transformer, and various combinations of them. Among them, we combine ResNet with LSTM, GRU and Bi-GRU in parallel, and the experimental results are shown in Table 5.

**Table 5:** Comparison experiments.

Model	Accuracy	*F*1 *score*
LSTM	69.21%	0.462
GRU	72.32%	0.495
Bi-GRU	73.68%	0.523
Res Net	79.21%	0.562
Transformer	74.52%	0.538
Res Net + LSTM	80.22%	0.586
Res Net + GRU	82.15%	0.592
Res Net + Bi-GRU	86.21%	0.587
SE-Res Net + Transformer Encoder	90.23%	0.647

The experimental results show that among all the tested models, the one in which we parallelized Transformer with the SE-Res module exhibited the highest accuracy and F1 score, and this design not only improves the expressive power of the model, but also enables the model to learn the nuances in the data more efficiently, thus achieving the best classification performance. This parallel combination not only utilizes the powerful feature extraction capabilities of the SE-Res module and the TF-Attention module, effectively solves the gradient vanishing problem in the deep network through its internal residual learning mechanism, and enhances the learning of feature representations in both the time and frequency domains, but also takes advantage of the Transformer encoder’s property of establishing connections throughout the entire range of sequences, allowing the model to distinguish between different positional elements and different time steps representing different music segments.

In conclusion, the experimental results show that our proposed multimodal fusion structure can significantly enhance the model and provide valuable directions for future research.

## 5 Conclusion and limitation

### 5.1 Conclusion

Relying on Internet platforms and intelligent terminals, the digital music market has achieved rapid growth in the number of music releases and the number of digital music users. At the same time, competition among music streaming platforms has become increasingly fierce, with new features and services constantly launched among the various platforms to attract more users and improve user retention. The classification of a large number of music genres is conducive to the realization of personalized recommendation on music platforms, improve people’s satisfaction with songs pushed by music platforms, and promote the development of music market. In order to improve the accuracy of music type classification, we propose the TS-Resformer model, which mainly completes the following tasks:

(1)   In the data processing part, we propose an audio slicing method with different time durations. This method solves the problem that too long audio duration in the data set is not conducive to model training, and at the same time, by comparing the effects of different slicing durations on the accuracy and training time of music genre classification, we determine the optimal slicing duration and realize data enhancement.
(2)   In order to obtain comprehensive and effective music audio feature information and better realize music genre classification, we propose a parallel music genre classification model Res-Transformer based on residual network and Transformer Encoder, which improves the residual network instead of convolution operation, improves the ability of the network to extract features of Log Mel spectrum, and alleviates the problem of gradient disappearance. The model improves the feature extraction capability of the network for Log Mel spectrum by improving the residual network instead of the convolution operation, which alleviates the problem of gradient disappearance.
(3)   Aiming at the problem of insufficient feature extraction in the Res-Transformer model, which leads to the low classification accuracy of individual genre, the TS-Resformer model is established by integrating the time-frequency and channel attention mechanisms. The feature extraction layer of the model is designed to capture features from both time and frequency dimensions using filters of different scales, and the filter scales are designed for music genres with low accuracy rates of individual genres, so as to extract low-level music features from specific time and specific frequency. The output of the time-frequency convolution is used as the input of the time-frequency attention mechanism, and the time-frequency attention mechanism applicable to music classification is designed to assign weights to the extracted music features in the time-frequency dimension, which mitigates the problem of insufficient music feature extraction and the problem of low accuracy rate of classification of individual category genres. By fusing the residual block with the improved channel attention mechanism, the weighting of each channel of the time-frequency feature map is realized, which weakens the features that are not related to music classification and reduces the complexity of the global features after feature fusion.

### 5.2 Limitation

With the gradual expansion of the global music market, the task of music classification in the future should not only require higher classification accuracy, but also achieve non-exclusive music genre classification according to music styles. Therefore, there are still many aspects that could be improved in this paper:

(1)   The method of classifying music genres by deep learning is mainly to convert music into Log Mel spectrum like ordinary audio, and then use the feature extraction capability of convolution for images. It is necessary to further study effective music data processing methods based on music characteristics.
(2)   This paper is mainly based on single label classification, but in fact, some concerts have the characteristics of multiple genres, which cannot be simply classified into one class. In the future, it is necessary to establish or select multi-label data sets and further study music genre classification.

## Data Availability

Publicly available datasets were analyzed in this study. This data can be found at: https://github.com/mdeff/fma.
